# Challenges of P300 Modulation Using Transcranial Alternating Current Stimulation (tACS)

**DOI:** 10.3389/fpsyg.2019.00476

**Published:** 2019-03-05

**Authors:** Fabian Popp, Isa Dallmer-Zerbe, Alexandra Philipsen, Christoph S. Herrmann

**Affiliations:** ^1^Experimental Psychology Lab, Department of Psychology, European Medical School, Cluster for Excellence “Hearing for All”, Carl von Ossietzky University of Oldenburg, Oldenburg, Germany; ^2^Department of Psychiatry and Psychotherapy, Universität of Bonn, Bonn, Germany; ^3^Research Center Neurosensory Science, Carl von Ossietzky University of Oldenburg, Oldenburg, Germany

**Keywords:** transcranial alternating current stimulation, EEG, ERP, ERO, P300, P3b

## Abstract

The P300 component of the event-related potential (ERP) is a well investigated phenomenon in the human electroencephalogram (EEG) and has been related to stimulus processing and attentional mechanisms. Event-related oscillations (ERO) represent a potential mechanism responsible for generating the ERP. In particular, oscillatory activity in the delta and theta frequency range has been associated with the generation of the P300 component. Transcranial Alternating Current Stimulation (tACS) is capable of modulating oscillatory brain activity in a frequency-specific manner. In this study, we aimed to modulate P300 amplitude using tACS by stimulating the individual ERO involved in the generation of the P300 component. TACS was applied precisely in time to the target P300 occurring in a visual oddball task. In order to achieve an appropriate current distribution, we designed an electrode configuration consisting of two clusters of stimulation electrodes on central-parietal locations. We could not demonstrate a group difference in P300 amplitude after applying tACS in the stimulation condition (*N* = 17) vs. the sham condition (*N* = 11). TACS condition and sham condition did not differ regarding their reaction times in response to target stimuli or their event-related spectral perturbation (ERSP) at stimulation frequency. Although a significant influence of stimulation could not yet be revealed on a statistical level, we suggest that the proposed method of using tACS for modulating EROs merits further investigation. Modulation of the P300 component in the ERP could help to gain further insights in the role of EROs generating ERPs and the functional relevance of the P300 component. In this study, we propose a novel approach of applying tACS and provide advice on using tACS for the modulation of EROs.

## Introduction

The event-related potential (ERP) in the electroencephalogram (EEG) typically consists of early potentials such as the P100, N100, P200, and N200 components mainly involved in sensory processing. Depending on the task and the stimulus, late positive deflections, representing rather cognitive aspects of stimulus processing can occur additionally (Polich, 2007).

The P300 component is characterized by a large positive deflection (10 – 20 μV) occurring roughly 250 – 500 ms after stimulus onset (Polich, 2007). This widely investigated phenomenon has been described first by Sutton et al. (1965). Corresponding to the variety of experimental characteristics of the behavioral tasks, several large positive ERP components can be distinguished (Linden, 2005; Polich and Criado, 2006). A common paradigm to investigate the P300 component is the oddball task (Ritter and Vaughan, 1969), consisting of successively presented standard and target stimuli, intermitted by an inter-stimulus interval. Only the rarer occurring target or “oddball” stimulus requires a response of the participant. The oddball paradigm has been used in the visual (e.g., Ravden and Polich, 1999; Bledowski et al., 2004a,b; Saville et al., 2015) and the auditory domain (e.g., Duncan-Johnson and Donchin, 1977; Polich, 1986, 1987a,b; Verleger and Berg, 1991).

The P300 component is characterized by its amplitude and latency. P300 amplitude is defined as the voltage difference of a pre-stimulus baseline and the largest positive deflection of the ERP within the time range of 250 – 500 ms (Polich and Kok, 1995; Herrmann and Knight, 2001) and has been associated to the intensity of stimulus processing. A variety of task-related parameters have been found to influence P300 amplitude, such as attention to the stimulus, task relevance and task difficulty (Kok, 2001).

P300 latency is defined as the time interval from stimulus onset to the point of the largest deflection and has been linked to processing speed, corresponding to the time needed for detecting and evaluating a target stimulus (Kok, 2001; Polich, 2007). Several factors were identified influencing the P300 latency, such as the sensory domain of the stimulus, participant age and task conditions (Polich and Kok, 1995; Polich, 2007).

In an oddball task as described above, the component following the attended target stimulus is referred to as target P3 or P3b. It is characterized by a parieto-central scalp topography (Linden, 2005). Also, a component termed P3a has been identified in literature (Squires et al., 1975; Snyder and Hillyard, 1976). It appears after distractor stimuli that have been additionally introduced to the classical oddball paradigm or as response to novel stimuli (Polich and Kok, 1995). In contrast to the P3b component it has an earlier latency and a fronto-central scalp topography (Linden, 2005). Further, the possibility of an overlap of P3a and P3b components has been pointed out as a result to experimental manipulation (Brookhuis et al., 1981; Kok, 2001). In the present study we will neglect aspects of the P3a and will focus on the P3b component.

One approach of addressing the functional relevance of the P300 is the context-updating theory (Polich, 2007). According to this approach, the P300 is the neural correlate of a changing mental model as a response to altered experimental conditions. Thus, context updating can be subdivided into two basic cognitive processes, working memory and attention. Working memory is required to compare the current stimulus to the preceding stimuli. In case the current stimulus is deviating from the standard stimulus, attentional resources are allocated to the target stimulus.

There is an ongoing debate on the generation mechanisms of ERPs (Fell et al., 2004; Min et al., 2007). Several authors suggested that ERPs emerge as a result of a phase-reset of ongoing neural activity (Makeig et al., 2002; Klimesch et al., 2007). Ongoing brain signals represent a mixture of transient activity and sustained oscillatory activity (Jmail et al., 2011). Oscillatory activity can be classified by its frequency and includes in the human EEG typically delta (0 – 3.5 Hz), theta (4 – 7 Hz), alpha (8 – 12 Hz), beta (13 – 30 Hz) and gamma (30 – 80 Hz) frequency ranges. Each of these frequency bands is considered to contribute to one or more cognitive functions, depending on the involved brain area and the respective parameters of the oscillation amplitude, frequency, phase and coherence (Herrmann et al., 2016). Also, transitions between the frequency ranges are often indistinct. Brain oscillations are considered to represent local as well as long-range communication in the brain and have been linked to a variety of cognitive processes (Başar et al., 2001; Merker, 2013; Herrmann et al., 2016). An alternative explanation for the generation of ERPs, however, has also been proposed: the additive power model. It has been argued, that ERPs result from a specific activation of neural assemblies with fixed polarity and latency, independent of ongoing neural activity. Based on this model, the ERP is generated by a stimulus-related power increase adding up to an ERP (Jervis et al., 1983; Schroeder et al., 1995; Lopes da Silva, 1999). Min et al. (2007) provided evidence that the two approaches on the generation of ERPs are not necessarily exclusive, but could both be influential (Min et al., 2007).

Sensory stimuli that evoke ERPs also influence ongoing brain activity in various frequency domains, e.g., in the delta (Başar-Eroğlu et al., 1992), theta (Başar-Eroglu et al., 1991), alpha (e.g., Schürmann and Başar, 2001; Kasten and Herrmann, 2017) and gamma (Yordanova et al., 2002) frequency range. Because of the close relation of brain oscillations and ERP components, the ERP complex can be regarded as a superposition of the synchronized oscillatory activity (Herrmann et al., 2014). This phenomenon has been described as event-related oscillations (EROs).

On the other hand, activity in a certain frequency spectrum does not necessarily represent a sustained brain oscillation at that frequency range, but could stem from other sources like artifacts (Herrmann et al., 2014) or even transient activity in the time domain (Jones, 2016).

However, several studies were able to illustrate the relationship between oscillatory activity in the delta and theta frequency range and P300 complex in auditory and visual oddball tasks (see the extensive review of Güntekin and Başar, 2016). First, a frequency specific power increase was demonstrated for the delta (Schürmann et al., 1995; Kolev et al., 1997; Demiralp et al., 2001) and theta (Başar-Eroğlu et al., 1992; Demiralp et al., 2001) frequency range in the target compared to the non-target condition. Second, time-frequency components in the delta and theta frequency range occuring at the typical latency of the P300 component (Demiralp et al., 2001; Quiroga et al., 2001) were identified even on a single trial basis (Kolev et al., 1997) and matched the characteristic parietal scalp topography of the P300 (Demiralp et al., 1999). Even though previous research is correlational in nature, it has been hypothesized that oscillations in the delta and theta frequency ranges represent possible mechanisms for the generation of the P300 component (e.g., Yordanova and Kolev, 1998; Jones et al., 2006).

Besides other non-invasive brain stimulation (NIBS) methods [e.g., transcranial magnetic stimulation (TMS, Thut et al., 2012) or visual flicker (Notbohm et al., 2016)], transcranial alternating current stimulation (tACS) has been proposed to modulate oscillatory brain activity in a frequency specific manner (see Herrmann et al., 2013; Reato et al., 2013 and Herrmann and Strüber, 2017 for review).

Several studies revealed electrophysiological and behavioral effects after tACS in the delta and theta frequency range. Vosskuhl et al. (2015) showed increased theta amplitude and improved working memory performance after tACS in the theta range. Wischnewski and Schutter (2017) tried to enhance evoked delta- and theta activity using tACS. Although the authors failed to demonstrate an effect of tACS on evoked theta activity, EEG delta power was decreased after stimulation (Wischnewski and Schutter, 2017). However, in that study, the authors did not tune the stimulation to the intrinsic frequency and phase of the event-related oscillation. Thus, the phase of the tACS is likely randomly distributed relative to the phase of the evoked oscillation. It is even possible that the stimulated sinusoidal waveform was applied in an anti-phasic manner to the evoked oscillation. This might have impaired the phase-reset of the event-related oscillation. This in turn might have led to a disruption of the evoked oscillation, possibly explaining the reported power decrease in the delta frequency range. A recent study by Pahor and Jaušovec (2018) illustrated enhanced theta amplitude in resting EEG after tACS in the theta frequency range (Pahor and Jaušovec, 2018). Moreover, Kasten et al. (2016) demonstrated that tACS aftereffects in the alpha frequency band are present up to 70 min after stimulation.

Given the fact that the time frequency decomposition of the P300 reveals peaks in the delta and theta frequency range (Kolev et al., 1997), we hypothesize that tACS in this frequency range should enhance P300 amplitude. Further, we hypothesized an improvement in task performance related to a P300 amplitude increase. Task performance is represented by the measures response accuracy and reaction times. An increase in accuracy was expected, as it has been shown that an increase in P300 amplitude can be accompanied by an improvement of task-related accuracy induced by methylphenidate in children suffering from attention deficit and hyperactivity disorder (ADHD, Jonkman et al., 2007). Schoenberg et al. (2014) revealed an amelioration of task-related attention in ADHD patients in association with a P300 amplitude increase. Even though reaction times represent a rather indirect measure of attention, we thus hypothesized a decrease in reaction times in conjunction with the expected P300 amplitude increase.

Previous studies reported to have influenced induced (Pahor and Jaušovec, 2016; Kasten and Herrmann, 2017) and evoked oscillations (Wischnewski and Schutter, 2017) with tACS. This study is -to our knowledge- the first using tACS to directly target the P300 component.

A potential modulation of the P300 component using tACS is promising from different perspectives. First, a P300 modulation caused by tACS in the delta and theta frequency ranges would further strengthen the concept of event-related oscillations. Also, a successful manipulation of the P300 amplitude by tACS would constitute a novel approach of applying tACS. This in turn could be used to further investigate the functional relevance of this ERP component.

## Materials and Methods

### Participants

Twenty-nine volunteers participated in the study after giving their written consent. All participants were students at the Carl von Ossietzky University Oldenburg and received monetary compensation. A questionnaire ensured that the participants were free of medication and were not suffering from psychiatric or neurological diseases. Participants were right handed according to the Edinburgh handedness scale (Oldfield, 1971). One participant was excluded due to technical issues during the recording. Hence, 28 participants (14 female, at an average age of 24.4 years, *SD* = 2.96) remained for the analysis (17 in stimulation group, 11 in sham group). The assignment to stimulation or sham condition was random. The study was designed and performed according to the declaration of Helsinki and approved by the local ethics committee of the Carl von Ossietzky University Oldenburg (“Kommission für Forschungsfolgenabschätzung und Ethik”).

### EEG Recording

**FIGURE 1:**
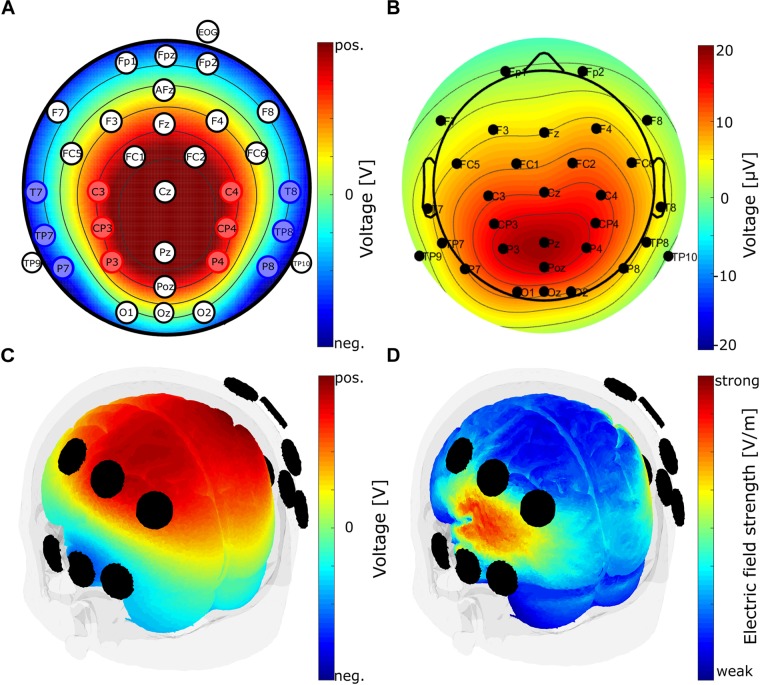
Electrode configuration and finite element modeling of the electrode clusters. **(A)** Electrode positions of the EEG electrodes according to the international 10-10 system. Red and blue positions indicate electrodes used for tACS in block 2 of the experiment. Electrodes used for tACS are arranged in two electrode clusters, red and blue. Each cluster is used as one tACS electrode (alternatingly anode or cathode). Color map shows voltage topography on the scalp resulting from the two clusters of stimulation electrodes. Depicted is the case of the medial cluster being the anode and the lateral cluster being the cathode. TACS amplitude was set to 1 mA peak-to-peak. Voltage was adjusted automatically by the stimulation device according to individual electrode impedances. **(B)** P300 component topography of the EEG baseline block, averaged over subjects at the subject’s individual P300 peak latency. **(C)** Voltage topography distribution on brain surface obtained from finite element modeling using the mentioned stimulation electrode clusters and a current strength of 1 mA peak-to-peak. **(D)** Electric field distribution on brain surface obtained from finite element modeling with the same parameters as in **(C)**.

Electroencephalogram was measured using 34 electrodes positioned according to the international 10-10 system (Figure 1A). The signal was recorded by a BrainAmp amplifier (Brain Products, Gilching, Germany). Sintered Ag/AgCl electrodes were used and mounted on an elastic cap (EasyCap GmbH, Herrsching, Germany). The reference electrode was placed on position FPz and the ground electrode on position AFz. A vertical Electrooculogram (EOG) was recorded by an electrode placed below the participant’s right eye. Electrode impedances were kept below 10 kΩ. The range of the amplifier was ±3.2768 mV with a resolution of 0.1 μV and a sampling rate of 1000 Hz. The EEG measurement was conducted in an electrically shielded room.

The experiment consisted of three blocks. EEG was recorded during the whole course of the experiment. Additionally, a photo sensor was attached to the computer monitor. The signal was used to control precise stimulus timing after the experiment.

### Electrical Stimulation

Electrical stimulation was administered in experimental block 2 using a battery-operated stimulation device (NeuroConn, Ilmenau, Germany). Stimulation intensity was set to 1 mA peak-to-peak. Stimulation started with a fade-in of 10 s and ended with a fade-out of 10 s and lasted for 20 min in total. Stimulation electrode impedances were kept below 10 kΩ. Stimulation frequency was determined for each participant individually (see Procedure) and kept in the delta or theta frequency range (1 – 7.5 Hz). For individual stimulation frequency of each participant, see Supplementary Material. TACS was administered using 2 × 6 temporal-parietal electrodes arranged as two clusters of EEG electrodes (Figure 1A). One electrode cluster was placed medially (C3, C4, CP3, CP4, P3, and P4) and the other one laterally (T7, T8, TP7, TP8, P7, and P8). Each cluster served alternatingly as anode or cathode. Participants in the sham condition received only 20 s of stimulation, consisting of 10 s fade-in and 10 s fade-out. This was performed in order to trigger the sensation of tACS. No further electrical stimulation was administered in the sham group for the rest of the experiment.

Stimulation electrodes were selected making use of the Helmholtz reciprocity principle (Malmivuo and Plonsey, 1995). If there are one or more current sources in the brain, they will result in a voltage distribution on the scalp. The Helmholtz principle states that, if the identical voltage distribution is externally applied to the scalp, the resulting current flow inside the brain is similar in the location of the original sources. The current flow is only similar but not identical, since the direction of current flow is the same as that of the original sources, but the intensity may be different. This limitation can be neglected in our case, since the intensity oscillates between a positive and a negative value anyway. In addition, due to the ambiguity of the inverse problem, the Helmholtz reciprocity holds for all possible source locations that would have resulted in the same voltage distribution on the scalp. This, however, is not a limitation for our purpose, since at least those sources that in fact have led to the observed voltage distribution will receive current flow in the correct direction.

In order to identify two appropriate electrode clusters, prior to the experiment, the topography of the P300 component emerging from the target stimulus presentation was inspected (Figure 1B). Secondly, stimulation electrodes were arranged in a way that the voltage distribution emerging from the used electrode clusters matched the P300 component topography (Figure 1A). As the two topographies are quite similar, it can be inferred that the electric current flow resulting from the electric stimulation is located in the areas being involved in the generation of the P300 component.

In order to validate these assumptions, a finite element model was used to estimate the electric field and the voltage distribution in the brain emerging from the stimulation using these twelve electrodes. Modeling was performed using the ROAST toolbox (Huang et al., 2017) with the above mentioned stimulation parameters, stimulation electrode clusters and a MNI-152 standard head (Grabner et al., 2006). Figure 1C illustrates the estimated voltage distribution on brain surface. Figure 1D shows that the maximum of the electric field can be found in parietal and temporal cortices. The pattern of current flow nicely resembles the pattern of generators that have been identified for generation of the P300 by Bledowski et al. (2004b). The authors employed a source localization approach by means of functional magnetic resonance imaging (fMRI) exploring the neural sources of the P300 component. Three pairs of bilaterally placed regional sources in parietal and temporal cortices were identified (Bledowski et al., 2004b). Thus, it can be inferred that the applied stimulation leads to an electric field in the areas involved in P300 generation. However, since modeling was performed using a MNI standard brain, individual brain anatomy could lead to differences in the location of the electric field. This in turn could individually affect the efficacy of the applied electrode montage.

As the electrode of the two clusters were used for stimulation in block 2, EEG was only recorded from the remaining electrodes during stimulation. Stimulation electrodes were connected to the stimulator before block 2 and reconnected to the EEG amplifier after block 2.

### Procedure

The experiment consisted of three consecutive blocks separated by breaks of about five minutes. Participants performed a visual oddball task in all three blocks. TACS or sham stimulation was administered only in the second block. EEG was recorded throughout the experiment. The task was controlled using Presentation (Version 18.01, Neurobehavioral Systems Inc., Albany, CA, United States).

**FIGURE 2:**
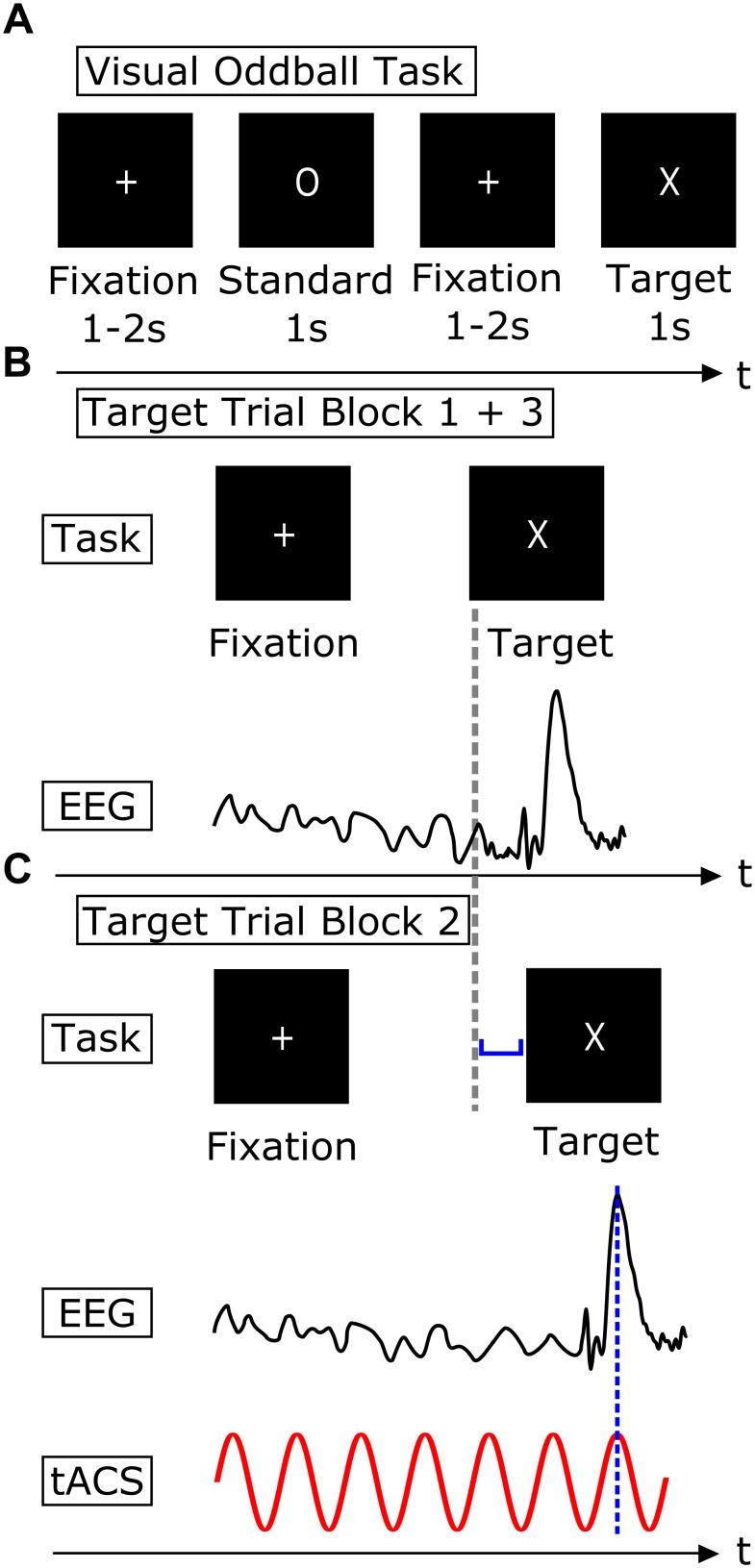
Timing of visual and electrical stimulation. **(A)** Visual Oddball Task. The task consisted of two different visual stimuli, a standard stimulus (“O”) and a target stimulus (“X”). Subjects were instructed to press a button whenever a target stimulus appeared on the screen and refrain from pressing the button whenever a standard stimulus was shown. Stimuli were presented for 1000 ms. Stimulus presentation was interrupted by an inter-stimulus interval which was randomly varied between 1000 and 2000 ms. **(B)** Schematic procedure of a single target trial in experimental block 1 and 3. Visual presentation of the target stimulus (upper row) and EEG with P300 component following the target stimulus (lower row). **(C)** Schematic procedure of a single target trial in experimental block 2. Target stimulus onset is adjusted (blue bracket) in order to ensure an overlap of the P300 peak and a peak of the sinusoidal tACS, indicated by the dashed blue line.

Participants had to press a button as fast as possible whenever a target stimulus (“X”) was shown and were instructed not to press when a standard stimulus was presented (“O”). Target stimuli appeared in 25% of the trials. In between trials a fixation cross was shown. Stimuli were presented for 1000 ms and inter-stimulus intervals randomly varied between 1000 and 2000 ms (Figure 2A). 400 experimental trials were presented in block 1 and 3, respectively, adding up to a block duration of about 16.6 min. Block 2 lasted 20 min and comprised of 480 trials. This resulted in 100 target trials in block 1 and 3 and 120 target trials in block 2. The number of target trials was higher in the stimulation in order to completely exhaust the 20 min of authorized stimulation length.

The stimulation was tailored individually using two parameters. First, each participant was stimulated at the frequency that contributed strongest to generation of his or her individual P300 component. Second, the individual latency of the P300 peak was used to adjust stimulus presentation to ensure temporal alignment of the peak of the tACS sinus and the P300 peak (Figure 2B,C).

Hence, a reasonable approximation of the individual P300 latency and frequency of this component was necessary. This was accomplished by a preliminary analysis of the EEG baseline signal of the first block of the experiment. This preliminary analysis was performed during the break between block 1 and 2.

**FIGURE 3:**
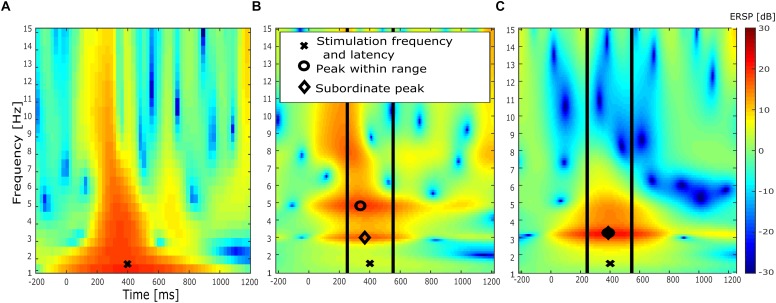
ERSP analysis of exemplary subject. **(A)** ERSP of EEG baseline block (block 1) after preliminary analysis during experiment with a frequency resolution of 0.5 Hz. “X” indicates maximum ERSP value. The frequency at which this value occurred was used as stimulation frequency. **(B)** ERSP of EEG baseline block (block 1) after elaborated EEG preprocessing and with a higher frequency resolution of 0.14 Hz. Symbols mark respective peak of three separate analysis approaches. “X” indicates stimulation parameters determined in the preliminary analysis shown in **(A)**. The first analysis compared the ERSP value at the stimulation parameters determined in **(A)**, marked by “X.” The second analysis is marked by the circle, indicating the maximum ERSP value within a time range of previously determined P300 latency ± 150 ms (vertical lines). Finally, the diamond marks ERSP peak located closest to stimulation frequency within the same time range. **(C)** ERSP of post-stimulation EEG block (block 3) after elaborated EEG preprocessing.

First, EEG data of experimental block 1 was high-pass filtered at 0.5 Hz and low-pass filtered at 20 Hz. Then, data epochs of -3000 to 4000 ms around target presentation were created and a baseline correction of -50 ms to stimulus onset was performed. Subsequently the grand-average ERP following the target stimuli was plotted and the latency of the P300 peak was determined. P300 latencies were in a pre-defined range of 300 to 650 ms. In order to identify the frequency contributing strongest to the P300 component, event-related spectral perturbation (ERSP, Delorme and Makeig, 2004) was computed (Figure 3A). Therefore, 3 cycle Morlet wavelets were used, resulting in a frequency resolution of 0.5 Hz. Center frequencies were ranging from 1.5 up to 20 Hz. Then, in a range of ±150 ms around the P300 peak latency, the maximum ERSP of the time frequency decomposition was determined and its frequency was used as stimulation frequency. The peak latency of the P300 component was used to adjust the target presentation to the effect that the P300 peak and the stimulation peak would concur. This simultaneity was achieved by adjusting the inter-stimulus interval. Initially, the time points of the sinusoidal peaks were computed for the respective individual stimulation frequency. The output trigger of the stimulation device was used to identify zero-crossings of the sine function. Using these two pieces of information, the peaks of the sine function could be determined precisely in time. The onset of the target stimulus presentation was shifted by the difference of the next upcoming stimulation peak and the individual P300 latency (Figure 2C). This ensured the coincidence of the peak of the individual stimulation frequency and the peak of the P300 component. Despite the manipulation of the inter-stimulus interval, the interval was kept in block 2 as in both other experimental blocks between 1000 and 2000 ms.

Individual P300 latency and amplitude was also determined for the sham group. Target stimulus presentation was adjusted according to the individual P300 latency just as in the tACS group.

After the experiment participants filled out a questionnaire assessing adverse effects of the stimulation. The questionnaire was a translated version of an existing questionnaire on adverse effects in transcranial electric stimulation (TES; Brunoni et al., 2011). Different possible adverse effects were rated on a scale from 1 to 4 (1 – none, 2 – mild, 3 – moderate, 4 – severe). Further, participants reported whether they attributed this effect to the stimulation (1 – no, 2 – remote, 3 – probable, 4 – definite).

Participants were also instructed to guess, whether they had been in the stimulation condition, or not. Finally, participants were informed about their experimental condition and the scientific purpose of the study.

### Data Analysis

Data Analysis was performed using self-written scripts in MATLAB 2016a (The MathWorks Inc., Natick, MA, United States) with functions of the EEGLAB toolbox, version 14.0.0b (Delorme and Makeig, 2004). Filtering was performed using the EEGLAB function *pop_eegfiltnew*, which applies a Hamming windowed sinc FIR Filter. For statistical analysis, the R software package was used, version 3.3.0 (R Foundation for Statistical Computing, Vienna Austria).

### Behavioral Data

Target trials with a button press after target stimulus presentation were classified as “hit trials.” Target trials in which the participant omitted the button press were classified as “miss trials.” Standard trials with no button press after standard stimulus presentation were classified as “correct omissions.” Standard trials with an erroneous button press after standard stimulus presentation were labeled as “false positive trials.”

Standard trials were not further analyzed. Reaction times were computed for target trials. Target trials identified as miss trials and target trials with reaction times smaller than 200 ms and larger than 1000 ms were excluded from further analysis. Moreover, the mean of the reaction times of all target trials of each subject were computed. All trials with reaction times greater than 2 standard deviations were excluded. This allowed an individual exclusion of outlier trials.

### EEG

Electroencephalogram data of block 2 was not analyzed, as tACS in a stimulation intensity of 1 mA causes high-amplitude tACS artifacts in the range of several mV (Helfrich et al., 2014).

Electroencephalogram data was high-pass filtered at 0.5 Hz and low-pass filtered at 20 Hz. Epochs containing values > 1000 μV were excluded. Then, an Independent Component Analysis (ICA) was performed. Independent components were visually inspected and components containing ocular artifacts were rejected before back-projection. After the ICA back-projection, epochs in which the difference between minima and maxima exceeded 200 μV were rejected. Epochs were created 3000 ms pre- and 4000 ms after target presentation. EEG epochs of the reaction time outlier trials were excluded. Baseline correction on each epoch was applied using a baseline of -200 ms to stimulus onset.

The target ERP was computed by averaging all target trials remaining in the analysis. Maximum P300 amplitude was compared amongst the following 4 parietal electrodes: P3, P4, Pz, and POz. EEG data of the electrode with the largest P300 peak amplitude was used for further analysis. This was implemented to compensate for a possible slight lateralization of the P300 component.

P300 component amplitude values were determined using the maximum amplitude value in a time window of to 400 to 600 ms after target stimulus onset. The latencies of these maximum amplitudes were used as P300 peak latencies. In addition to the absolute amplitude and latency values, relative changes of amplitude and latency were computed. This was implemented using the absolute values of the baseline block and the post-stimulation block (Equation 1).

(1) $$\mathrm{relative change}=\left(\frac{\left(\mathrm{block}3-\mathrm{block}1\right)}{\mathrm{block}1}\right)*100$$

In order to detect event-related power changes, ERSP was computed. 5 cycle Morlet wavelets were used for the wavelet transform with center frequencies ranging from 1 to 15 Hz in continuous steps of 0.1414 Hz. For the purpose of evaluating potential differences regarding the ERSP between tACS and sham group, several analyses of the ERSP were performed. First, ERSP was compared at the individual stimulation frequency ±1 Hz. This test was implemented in order to detect a potential power increase in the stimulated frequency band. This was done, as a power increase due to tACS stimulation is usually expected close to or at the stimulation frequency (Herrmann et al., 2013). Second, ERSP at stimulation frequency ±1 Hz and ±150 ms around the P300 peak latency was compared. This analysis aimed at detecting a potential power increase in the stimulated frequency band only in the time window of P300 occurrence. This is sensible, as the stimulation peak concurred with the P300 component. Therefore, a time-specific power increase is conceivable and was hypothesized above. The third analysis was conducted to deal with potential multiple peaks in the frequency and time range of the P300 component (as in the subject depicted in Figure 3B). Therefore, a time range of 150 ms around the determined P300 latency was defined. Then, the peak closest to the stimulation frequency was taken and the ERSP value of this peak was compared between the baseline block and the post-stimulation block, indicated by a diamond in Figure 3B,C.

This was done as a power increase is expected in the peak closer to the stimulation frequency because of the frequency specificity of tACS. Further, the relative changes of the ERSP were determined using Equation 1 and was compared on group level.

Finally, spectral power analyses were performed in order to examine potential event-unrelated power changes in the stimulated frequency bands.

Therefore, Fast Fourier transforms (FFTs) were computed on 1 s epochs of the data, including target and standard trials. Relative change (see Equation 1) of power from baseline block 1 to post-stimulation block 3 was computed, accounting for inter-individual power differences. Four different comparisons were performed on relative power change.

(1) In order to assess whether the stimulation had an influence on the relative power change in the delta and theta frequency band (0.5 – 7.5 Hz), relative power change was compared between groups. (2) Relative power change of the tACS group only in the delta frequency band was compared to the sham group (0.5 – 3.5 Hz). (3) Similarly, relative power change only in the theta frequency band (4 – 7.5 Hz) was compared between groups. Finally, two sub-groups were formed: (4) Subjects who received stimulation in the delta frequency range (*N* = 12) and (5) subjects who received stimulation in the theta frequency range (*N* = 5). Then, spectral power in the respective frequency band for each sub-group was compared against the sham group.

## Results

### Behavioral Data

A mixed factorial ANOVA was computed in order to explore potential differences of reaction times between groups and between experimental blocks. It included the within-subject factor *block* (3 levels, baseline block, stimulation block, post-stimulation block) and the between-subjects factor *group* (2 levels, tACS and sham). Further, *post hoc t*-tests were performed in order to unveil differences in reaction times between pairs of blocks. Lastly, Pearson’s product-moment correlation was computed for reaction times and P300 latencies. For all statistical tests a significance level of α = 0.05 was applied. In case Mauchly’s Test of Sphericity indicated a violation of sphericity, Greenhouse-Geisser correction was applied when Greenhouse-Geisser ε was <0.75, otherwise Huynh-Feldt correction was performed (Girden, 1992). The mixed factorial ANOVA on the reaction times did not yield a significant difference in reaction times for the factor *group F*_1,26_ = 0.788, *p* = 0.383, η^2^ = 0.028, *M_stim_* = 411.84 ms, *SD_stim_* = 60.83 ms, *M_sham_* = 393.92 ms, *SD_sham_* = 54.46 ms and the interaction *group x block F_1.66,43.08_* = 0.474, *p* = 0.590, η^2^ = 0.001 (both Huynh-Feldt corrected). A significant effect for the factor block could be demonstrated *F_1.66,43.08_* = 6.598, *p* = 0.005, η^2^ = 0.017, *M_Block1_* = 413.14 ms, *SD_Block1_* = 59.82 ms, *M_Block2_* = 404.72 ms, *SD_Block2_* = 57.68 ms, *M_Block3_* = 396.54 ms, *SD_Block3_* = 57.49 ms.

**FIGURE 4:**
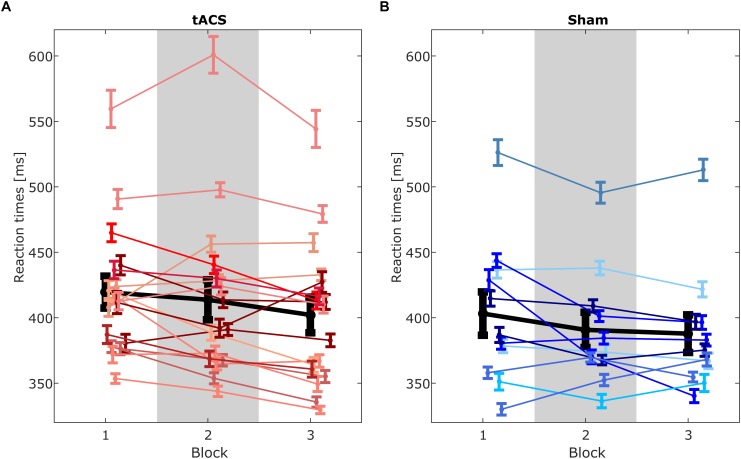
Reaction times of single subjects for **(A)** tACS group and **(B)** sham group. Each line represents means of reaction times of a single subject for each block. Lines linking block means illustrate changes between blocks. Error bars depict standard error of the mean. Group means shown in black. Gray rectangle marks block 2 in which stimulation was applied.

**FIGURE 5:**
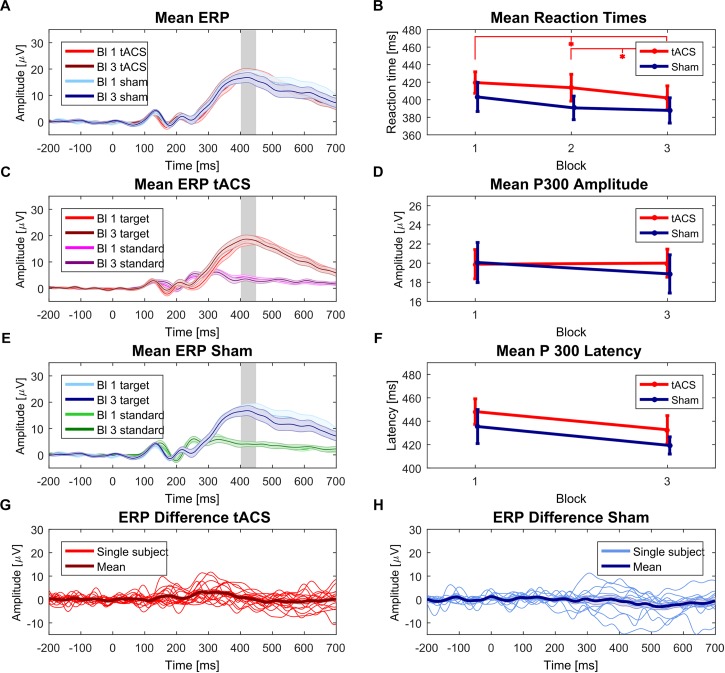
Behavioral and EEG results. **(A)** Mean ERPs of tACS and sham group pre and post stimulation. Shaded areas and error bars in all plots depict the respective standard error of the mean. Gray rectangle in **(A,C,E)** indicates that amplitude comparison was performed at individual P300 amplitude peak. **(B)** Reaction times of tACS and sham condition for each experimental block (baseline, stimulation block and post stimulation block). Asterisks indicate *p* < 0.05 for one-sided paired sample *t*-tests between baseline and post-stimulation block and between stimulation block and post stimulation block of the tACS group. **(C)** Mean ERPs of tACS group before and after stimulation. Standard trials displayed for comparison. **(D)** Mean P300 amplitude of the tACS and sham group before and after stimulation. **(E)** Mean ERP of sham group before and after stimulation. Standard trials displayed for comparison. **(F)** Mean P300 latency of tACS and sham condition before and after stimulation. **(G)** Individual ERP differences (Block 3 – Block 1) of target trials of the tACS group. Positive values indicate amplitude increase after stimulation block. **(H)** Individual ERP differences (Block 3 – Block 1) of the sham group.

In order to explore the difference in reaction times between blocks more closely, one-sided *post hoc t*-test were performed. The *post hoc t*-test on reaction times were computed one-sided, as a decrease of reaction times due to the stimulation was hypothesized. All p-values of *post hoc t*-tests were corrected for multiple comparisons using the FDR correction method by Benjamini and Hochberg (1995) implemented in the function *p.adjust* of the R software package. Individual reaction times are shown in Figure 4. A paired-sample *t*-test of the reaction times of the EEG baseline block and the stimulation block revealed significantly faster responses in the stimulation block (*t_27_* = 1.806, *p* = 0.041, *Cohen’s d* = 0.341, one-sided). Also, reaction times were significantly lower in the post-stimulation block compared to the stimulation block (*t_27_* = 2.449, *p* = 0.016, *Cohen’s d* = 0.463, one-sided). Finally, a one-sided paired-sample *t*-test of the EEG baseline block and the post-stimulation block yielded significantly faster reaction times in the post-stimulation block compared to the baseline block (*t_27_* = 3.107, *p* = 0.007, *Cohen’s d* = 0.587, one-sided). Mean reaction times are depicted in Figure 5B.

Participants of both groups achieved high levels of accuracy in the oddball task. Percentage of hit trials in the tACS group (*M_Block1_* = 98.91%, *SD_Block1_* = 3.69%, *M_Block2_* = 97.77%, SD_Block2_ = 8.10%, *M_Block3_* = 97.96%, *SD_Block3_* = 7.43%) and sham group (*M_Block1_* = 99.51%, SD_Block1_ = 1.32%, *M_Block2_* = 99.78%, SD_Block2_ = 0.53%, *M_Block3_* = 99.74%, *SD_Block3_* = 0.45%) were similarly high, as expected because of the simplicity of the task. Further, the percentage of false positive trials was low in the tACS group (*M_Block1_* = 0.10%, *SD_Block1_* = 0.19%, *M_Block2_* = 0.12%, *SD_Block2_* = 0.24%, *M_Block3_* = 0.24%, *SD_Block3_* = 0.41%) and the sham group (*M_Block1_* = 0.09%, *SD_Block1_* = 0.15%, *M_Block2_* = 0.13%, SD_Block2_ = 0.23%, *M_Block3_* = 0.15%, SD_Block3_ = 0.18%). Accuracy measures were not statistically tested, because of apparent ceiling effects in both experimental groups. In order to examine the relation of reaction times and P300 latency, Pearson’s product-moment correlation of these two measures was computed for experimental blocks 1 and 3. Correlations were computed one-sided, as only positive correlations were expected. P300 latency and reaction times were significantly correlated in the tACS group in block 1, *r*(15) = 0.693, *p* = 0.001 and block 3, *r*(15) = 0.729, *p* < 0.001. Similarly, high correlations were found for P300 latency and reaction times in the sham group in block 1, *r*(9) = 0.875, *p* < 0.001 and block 3, *r*(9) = 0.842, *p* < 0.001. Thus, a close relation of P300 latencies and reaction times were demonstrated for both stimulation groups before and after the stimulation and therefore replicate this previously reported phenomenon (see Kok, 2001; Polich, 2007).

The responses to the adverse effects questionnaire revealed highest ratings for *trouble concentrating* (67.9%), *tiredness* (64.3%), and *tingling* (46.4%). The tACS group declared significantly higher values for *scalp pain* than the sham group (*p* = 0.020, two-sided, *Cohen’s d* = 1.216). Also, the attribution of this factor to the stimulation was significantly higher in the tACS group compared to sham (*p* = 0.019, two-sided *Cohen’s d* = 1.228). However, the rating of *scalp pain* was relatively low (stim: *M* = 1.47, sham: *M* = 1), indicating the mild characteristic of this factor. All other factors did not differ between groups (*p* > 0.12). The answer to the question whether the participants believed to be in the stimulation condition revealed similar answers (stim: 76.47%, sham: 72.73%). Fisher’s Exact test for Count Data revealed no significant difference between groups (OR = 1.210, *p* = 1). This indicates that participants were not aware of the stimulation condition, even though the rating of the scalp pain differed between groups.

### EEG

In order to determine P300 amplitude and latency, ERPs were computed for each experimental block, stimulation condition and participant. Grand-average ERPs are shown in Figure 5A,C,E.

For the P300 peak amplitudes and latencies, two mixed factorial ANOVAs were performed. The ANOVA for the P300 amplitude values showed no significant effect of the factors *group* (*F*_1,26_ = 0.041, *p* = 0.841, η^2^= 0.001), *block (F*_1,26_ = 0.254, *p* = 0.619, η^2^ = 0.001), or the interaction *group x block (F*_1,26_ = 0.631, *p* = 0.434, η^2^ = 0.003). Mean P300 amplitudes for the tACS and sham condition are shown in Figure 5D. Individual amplitude plots are included in the Supplementary Material. ERP differences for each group are shown in Figure 5G,H.

The ANOVA for the peak latencies did not yield any significant differences for the factors *group (F*_1,26_ = 0.758, *p* = 0.392, η^2^ = 0.022), *block* (*F*_1,26_ = 4.110, *p* = 0.053, η^2^ = 0.033), or the interaction *group x block (F*_1,26_ = 0.002, *p* = 0.968, η^2^ < 0.001). Mean P300 latencies for each condition before and after stimulation are displayed in Figure 5F.

In order to determine whether the mismatch of stimulation parameters have an influence on the P300 amplitude after stimulation, frequency mismatch (*M* = 3.84 Hz, *SD* = 2.93) and time mismatch (*M* = 146.00 ms, *SD* = 92.71) for the tACS group was analyzed. Frequency mismatch and time mismatch were calculated using the differences of the values determined in the preliminary analysis during the experiment and the values determined in the offline analysis. A linear regression was computed in order to predict P300 amplitude change based on mismatch of stimulation frequency and mismatch of stimulation time. The model failed to significantly predict P300 amplitude (*F*_2,14_ = 2.443, *p* < 0.123 with an *R*^2^ of 0.153) using the predictors frequency mismatch (β = -0.602, *p* = 0.057), time mismatch (β = 0.008, *p* = 0.423) and an intercept (β = 1.310, *p* = 0.287).

A mixed factorial ANOVA was computed comparing the ERSP at stimulation frequency ± 1 Hz. No significant differences were detected for the factors *group (F*_1,26_ = 2.756, *p* = 0.109, η^2^ = 0.093), block (*F*_1,26_ = 0.444, *p* = 0.511, η^2^ = 0.001), or the interaction *group x block (F*_1,26_ = 1.129, *p* = 0.298, η^2^ = 0.003). A further mixed factorial ANOVA was computed comparing the ERSP at stimulation frequency ± 1 Hz for ERSP values ± 150 ms around the P300 peak latency. Similarly, no significant effect for the factors *group (F*_1,26_ = 2.974, *p* = 0.096, η^2^ = 0.095), *block* (*F*_1,26_ = 0.0385, *p* = 0.540, η^2^ = 0.001), or the interaction *group x block (F*_1,26_ = 0.917, *p* = 0.347, η^2^ = 0.003) could be demonstrated. An independent samples *t*-test of the relative changes of the ERSP values at stimulation frequency ±1 Hz for ERSP values ±150 ms between groups was performed, but also did not reveal a significant group difference (*t*_26_ = 1.108, *p* = 0.278, one-sided). Lastly, ERSP values of the local peaks closest to the stimulation frequency were compared using a mixed factorial ANOVA. The factor *group (F*_1,26_ = 0.788, *p* = 0.383, η^2^ = 0.020) showed no significant difference. A significant difference was shown in the factor *block (F*_1,26_ = 5.388, *p* = 0.028, η^2^ = 0.063). No significant interaction of the factors *group x block (F*_1,26_ = 0.316, *p* = 0.578, η^2^ = 0.004) could be demonstrated. Different analyses of the ERSP could not reveal an interaction of stimulation condition and experimental block in the time range of the P300 component.

An independent samples *t*-test comparing the relative amplitude change in the entire stimulated frequency band (0.5 – 7.5 Hz) did not show significant differences between tACS group and sham group (*t_26_* = -1.766, *p* = 0. 223, two-sided). Similarly, no significant differences were found when comparing the relative change only in the delta frequency band of the tACS group against sham group (*t_25_* = -1.161, *p* = 0. 428, two-sided). The same applies for the theta frequency band (*t_26_* = -1.903, *p* = 0. 223, two-sided). Two independent samples *t*-tests were computed comparing subjects stimulated in the delta range and subjects stimulated in the theta range compared to sham.

No significant result could be demonstrated in the delta (*t_16_* = -1.270, *p* = 0.999, one-sided), nor in the theta subjects (*t_14_* = -3.657, *p* = 0.999, one-sided). *P*-values of the spectral power analysis were corrected using FDR correction (Benjamini and Hochberg, 1995). The comparisons did not yield significant results. We therefore infer that tACS did not result in significant amplitude changes in the stimulated frequency bands.

## Discussion

Following the concept of event-related oscillations, oscillatory activity in the delta and theta frequency range has been suggested to contribute to the generation of the P300 component (Başar-Eroğlu et al., 1992). In a novel approach, we aimed to enhance P300 amplitude using precisely timed transcranial alternating current stimulation. We could not demonstrate a significant increase of P300 amplitude in the stimulation condition compared to a sham condition. Also, event-related spectral perturbation was not altered in the tACS condition as compared to the sham condition. Consequently, we cannot provide evidence that the presented methodological approach of temporally aligning sensory stimuli to the phase of tACS is capable of influencing EROs at this stage.

Our goal was to use tACS at the frequency of the P300 in order to enhance the amplitude of the P300. We assumed that this was plausible, since it has been demonstrated that tACS is able to modulate brain oscillations – probably via entrainment (Herrmann et al., 2013; Herrmann and Strüber, 2017) and that ERPs might be generated by the phase-reset of ongoing oscillations. Therefore, we expected that our results would shed light onto the question whether ERPs are generated by a phase-reset or by additive power. Our experiment, however, yielded no significant results. Thus, we cannot rule out either of the two models of ERP generation. In addition, we have to question whether we were able to entrain ongoing brain oscillations in the current experiment in the first place. The correct way to demonstrate that brain oscillations are entrained by tACS would be by demonstrating an Arnold tongue (Pikovsky et al., 2003). While this has been done for simulations of tACS (Reato et al., 2010), this has not been achieved in human data. It requires to stimulate at multiple stimulation frequencies, as well as multiple stimulation intensities. If the stimulation parameters are within the Arnold tongue, the brain oscillation follows the tACS stimulation. If, however, the stimulation parameters are outside the Arnold tongue, the brain oscillation oscillates at its intrinsic frequency. The Arnold tongue is a triangular region around the individual frequency of a brain oscillation that becomes wider as stimulation intensity increases.

The above-mentioned procedure, however, would require to differentiate the tACS artifact from true brain oscillations. While such an artifact *reduction* is possible in principle (Helfrich et al., 2014), it has been argued that a complete *removal* is probably impossible (Noury et al., 2016). A recent approach suggests to contrast conditions during tACS, which should contain similar amounts of residual artifact (Kasten et al., 2018). This approach has demonstrated that tACS at individual alpha frequency is able to modulate the event-related desynchronization in response to visual stimuli.

It should be noted that entrainment can only explain the online effects of tACS. In simulations as well as animal experiments, entrainment stopped after the end of the stimulation (Reato et al., 2010). For an after effect of tACS to occur, synaptic plasticity has been proposed (Zaehle et al., 2010; Vossen et al., 2015; Sliva et al., 2018).

Even if EROs were involved in the generation of the P300 and, furthermore, if also tACS had been successful in the entrainment of ongoing oscillations in our current experiment, several factors might have influenced the hypothesized modulation of the P300 component in an unfavorable manner.

The presumably most important factor is a mismatch between the stimulation frequency and the “true” endogenous frequency of the oscillations contributing to the ERP. We hypothesize the mechanism of entrainment to be responsible for online tACS effects. According to the concept of the Arnold tongue, the effect of entrainment is dependent of the factors phase and intensity and frequency of the entraining oscillator (Pikovsky et al., 2003; Notbohm et al., 2016). In case of a large mismatch of the external and the endogenous oscillator, the endogenous oscillation will remain unaffected. During the experiment, a preliminary EEG analysis was conducted. This preliminary analysis lacked EEG artifact rejection and contained a time-frequency analysis with only a poor frequency resolution. A more elaborate EEG analysis including artifact rejection methods after the experiment revealed in some participants a mismatch of the chosen stimulation frequency and the frequency with the highest ERSP value.

Consequently, it cannot be excluded that the occurred frequency mismatch was too large and prevented the mechanism of entrainment. Further, it has been suggested that tACS after-effects might depend on the mismatch of the frequency of the endogenous brain oscillation and the frequency of the external stimulation frequency (Vossen et al., 2015; Stecher et al., 2017). Even though the performed linear regression failed to reveal that frequency mismatch or time mismatch can predict P300 amplitude change, a precise determination of the stimulation parameters is crucial for a successful modulation of brain activity using tACS.

A modulation of task performance also could not be demonstrated on a behavioral level. Reaction times decreased significantly over the course of the experiment but revealed no interaction with stimulation condition or time.

Several patterns of individual reaction times are present. Some participants show a continuous decrease of reaction times from block 1 to block 3. This behavior is likely due to familiarization of the task and probably not due to stimulation effects. Other participants show a decrease of reaction times in the second block and similarly low values in the third block or again an increase of reaction times in the third block. These patterns could be due to stimulation effects. However, the absence of an interaction effect of the factors *group* and *time* indicate a decrease of reaction times independent of the stimulation condition. The analyses of the behavioral measures of miss trials and false positive trials did not reveal further insights to a possible modulation, since ceiling effects occurred due to the relatively easy task. Thus, increased task difficulty or the implementation of a different task could possibly lead to behavioral task modulation, as P300 amplitude has been suggested to reflect intensity of stimulus processing (Kok, 2001).

Several authors reported individual factors that might lead to a high inter-individual variability of non-invasive transcranial brain stimulation, such as age, gender, skull shape and structure as well as emotional or physiological states of participants (Thut et al., 2017). Although experimental conditions were counter-balanced for the factors age and gender, other factors were not controlled for.

A recent study by Vöröslakos et al. (2018) suggests that stimulation intensities of 1 mA peak-to-peak as used in this study might be not sufficient to achieve stimulation effects. Further, Minarik et al. (2016) emphasized the importance of sample size in studies in the related field of transcranial direct current stimulation (tDCS, Minarik et al., 2016). Due to small or intermediate effect sizes, a rather large sample is required in order to reveal a statistically significant effect. The factor *stimulation* was implemented as a between-subject factor in this study, which results in lower statistical power compared to a within-subject design. Thus, a within-subject design might be more adequate when trying to unveil rather small statistical effects in small samples.

Despite the fact that we were not able to modulate the amplitude of the P300 component in our experiment, a method increasing the P300 amplitude could be of importance as a therapeutic intervention. An obvious application is the treatment of psychiatric diseases with an amplitude reduction of the P300 component, such as ADHD or schizophrenia (Mathalon et al., 2000; Philipsen et al., 2008; Woltering et al., 2013).

The current study points out the importance of a high resolution of the time frequency decomposition used to determine the stimulation parameters. The application of artifact rejection methods before time frequency decomposition could further improve the presented approach. Moreover, the difficulty of the behavioral task could be increased in order to detect potential changes in the accuracy of the response behavior.

The presented experimental setup included the application of tACS and precisely timed stimulus presentation which ensured the coincidence of the stimulation peak and the P300 component. Further, a novel configuration of stimulation electrodes was reported, inducing the maximum of the electric field in temporal and parietal cortices. These have been identified as potential sources of P300 component generation (Bledowski et al., 2004b).

Future studies need to clarify whether tACS is capable of modulating the amplitude of ERP components by taking into account the problems we have encountered during our study.

## Author Contributions

All authors conceived and design the study, revised the manuscript, and read and approved the submitted version. FP and ID-Z acquired the data. FP analyzed the data. FP and CH wrote the manuscript.

## Conflict of Interest Statement

CH has filed a patent application on brain stimulation and received honoraria as Editor from Elsevier Publishers, Amsterdam. The remaining authors declare that the research was conducted in the absence of any commercial or financial relationships that could be construed as a potential conflict of interest.
