# Supplementary material for: Challenges of P300 Modulation Using Transcranial Alternating Current Stimulation (tACS)
Source: Front Psychol. 2019 Mar 5;10:476. doi: 10.3389/fpsyg.2019.00476 (PMC6411790; doi:10.3389/fpsyg.2019.00476)
Supplement: Supplementary file 1 [file Data_Sheet_2.PDF]

Supplementary Material

Challenges of P300 modulation using Transcranial Alternating Current Stimulation (tACS)

Fabian Popp[1], Isa Dallmer-Zerbe[1], Alexandra Philipsen[2,3], Christoph S. Herrmann[1,4]*

[1]Experimental Psychology Lab, Department of Psychology, European Medical School, Cluster for Excellence “Hearing for All”, Carl von Ossietzky University, Oldenburg, Germany

[2]Faculty of Medicine and Health Sciences, Carl von Ossietzky University, Oldenburg, Germany

[3]Department of Psychiatry and Psychotherapy, Karl-Jaspers Hospital, Bad Zwischenahn, Germany

[4]Research Center Neurosensory Science, Carl von Ossietzky University, Oldenburg, Germany

*** Correspondence:**
Christoph S. Herrmann
Email: christoph.herrmann@uni-oldenburg.de
Tel.: +49 441 798 4936
Fax: +49 441 798 3865

Note on unequal sample sizes. Please note that unequal sample sizes are due to the fact that the reported mismatches of the preliminary analysis of block 1 during the experiment and the offline analysis after the experiment became apparent during the analysis. At first, equal sample sizes were measured (N = 11, each). After the mismatches became apparent, it was planned to exclude the subjects of the tACS group with a large mismatch. Then, additional subjects in the tACS group were measured to compensate for the excluded subjects. After analyzing the dataset, significant amplitude effects could not be found. At this point, it was decided to include the mismatch-participants as the mismatch is due to too coarse resolution of the preliminary time-frequency analysis. This leads to the sample size of N = 17 in the tACS group and N = 11 in the sham group.

Participant	Stimulation frequency [Hz]	Stimulation latency [ms]	Signal electrode (pre/post)
tACS 1	2.5	430	(P4/Pz)
tACS 2	2.5	423	(Pz/Pz)
tACS 3	3.5	457	(P4/P4)
tACS 4	7.5	388	(P3/Pz)
tACS 5	2.5	364	(P3/Pz)
tACS 6	1.5	626	(Pz/Pz)
tACS 7	6.5	430	(Pz/Pz)
tACS 8	2.5	416	(POz/POz)
tACS 9	4.5	499	(POz/POz)
tACS 10	2	451	(POz/POz)
tACS 11	1	453	(POz/POz)
tACS 12	3.5	476	(Pz/Pz)
tACS 13	7.5	404	(Pz/Pz)
tACS 14	3.5	455	(Pz/Pz)
tACS 15	2.5	538	(P3/POz)
tACS 16	1.5	400	(POz/POz)
tACS 17	4	420	(POz/POz)
Sham 1	6	405	(POz/POz)

Sham 2	2	445	(Pz/Pz)
Sham 3	1.5	427	(Pz/Pz)
Sham 4	1.5	346	(Pz/P3)
Sham 5	2	451	(Pz/Pz)
Sham 6	1.5	632	(Pz/P3)
Sham 7	2.5	429	(P3/P3)
Sham 8	2	404	(Pz/Pz)
Sham 9	5	371	(Pz/Pz)
Sham 10	1.5	431	(Pz/Pz)
Sham 11	1.5	391	(P3/Pz)

Supplementary Table 1. Stimulation parameters of each participant. Stimulation frequency indicates the frequency of stimulation in the tACS group and the determined value for the sham group. Stimulation latency states the latency to which the peak of the stimulation waveform is tuned in order to achieve a concurrency of tACS peak and P300 component. Signal electrode describes the EEG electrode in which the largest P300 amplitude was detected and thus was used for further analysis.

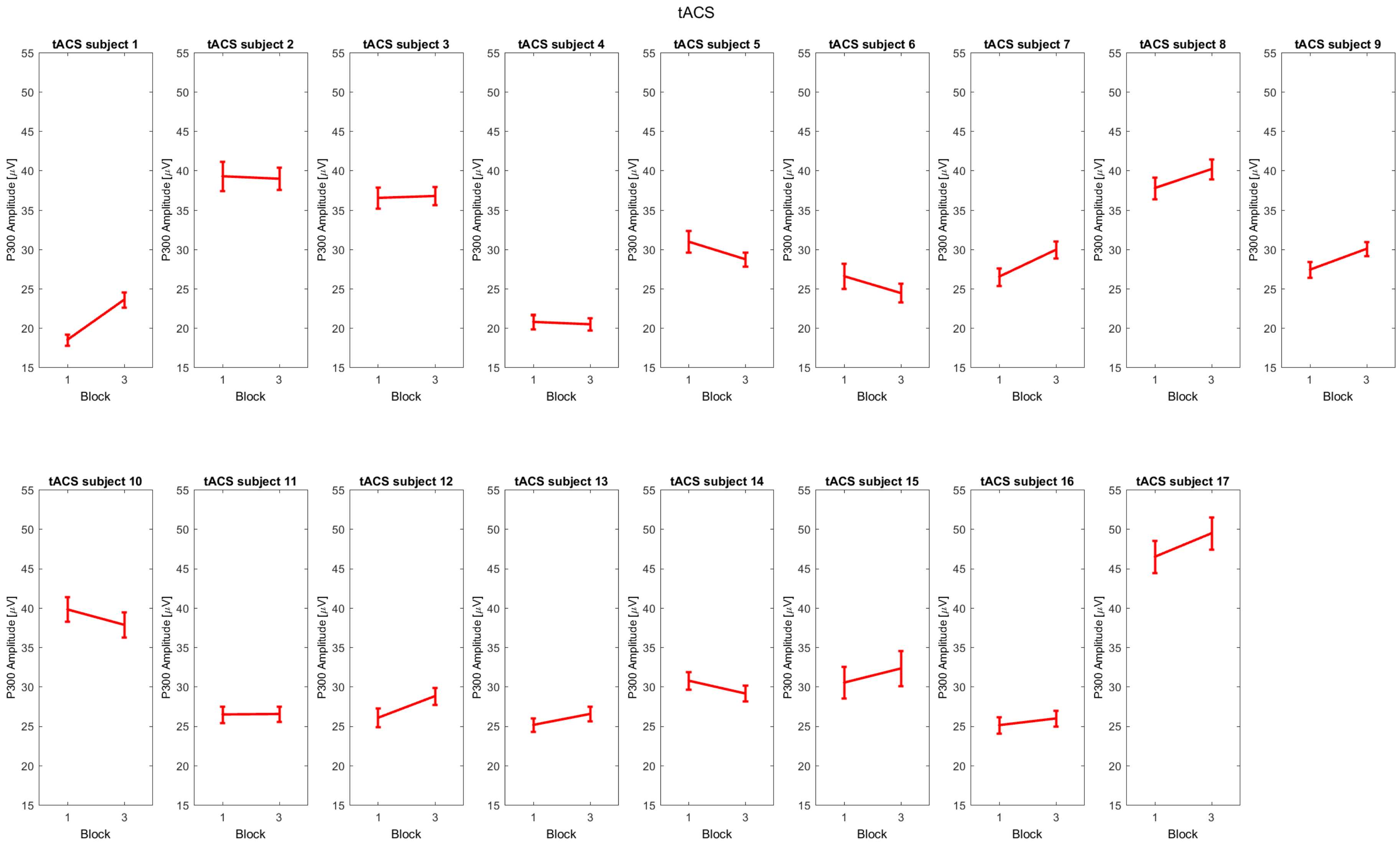

Supplementary Figure 1. Mean P300 amplitude values of the stimulation group for each subject and block. Error bars depict standard error of the mean.

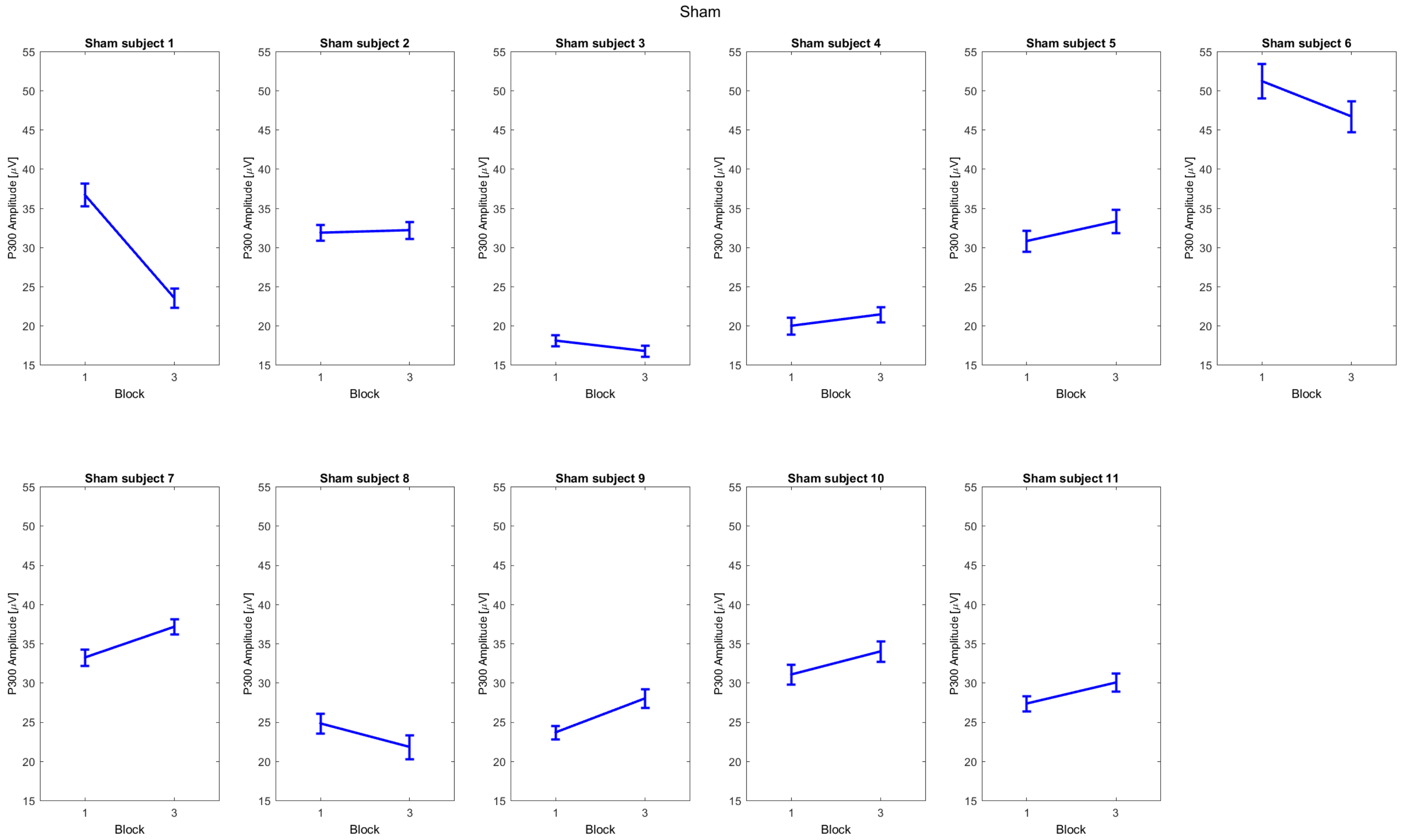

Supplementary Figure 2. Mean P300 amplitude values of the sham group for each subject and block. Error bars depict standard error of the mean.

	% of ratings > 1 all	% of ratings > 1 tACS	% of ratings > 1 Sham	M_{all}	M_{tACS}	M_{Sham}	SD_{all}	SD_{tACS}	SD_{Sham}
Headache	3.57	5.88	0	1.04	1.06	1.00	0.19	0.24	0
Neckpain	14.29	5.88	27.27	1.14	1.06	1.27	0.36	0.24	0.47
Scalppain	25	41.18	0	1.29	1.47	1.00	0.54	0.62	0
Tingling	46.43	52.94	36.36	1.57	1.53	1.64	0.74	0.52	1.03
Itching	22.22	25	18.18	1.30	1.25	1.36	0.61	0.45	0.81
Heat	32.14	41.18	18.18	1.68	1.88	1.36	1.09	1.17	0.92
Redness	14.82	6.25	27.27	1.19	1.13	1.27	0.48	0.50	0.47
Tiredness	64.29	64.71	63.64	2.21	2.24	2.18	1.13	1.15	1.11
Trouble concentrating	67.86	58.82	81.82	2.07	2.18	1.91	0.94	1.13	0.54
Mood swings	3.57	0	9.09	1.04	1.00	1.09	0.19	0	0.30
Other	0	0	0	1.00	1.00	1.00	0	0	0

Supplementary Table 2. Descriptive statistics of the adverse effects questionnaire. Each statistic is given for all participants and for each group separately. Percentage of ratings > 1 describes percentage of replies indicating the existence of the respective adverse effect. Remaining columns report means and standard deviations of each factor.
